# Synthesis and crystal structure of (1,10-phenanthroline-κ^2^ *N*,*N*′)[2-(1*H*-pyrazol-1-yl)phenyl-κ^2^ *N* ^2^,*C* ^1^]iridium(III) hexa­fluorido­phosphate with an unknown number of solvent mol­ecules

**DOI:** 10.1107/S2056989020005861

**Published:** 2020-05-05

**Authors:** Juxiang Zeng, Guodong Tang, Jun Qian

**Affiliations:** a Jiangsu Nursing Vocational College, Huaian 223300, Jiangsu Province, People’s Republic of China; b Jiangsu Key Laboratory for Chemistry of Low-Dimensional Materials, Huaiyin Normal University, Huaian 223300, Jiangsu Province, People’s Republic of China; c School of Chemistry and Chemical Engineering, Jiangsu University, Zhenjiang 212013, People’s Republic of China

**Keywords:** crystal structure, cyclo­metallated iridium complex, 1,10-phenanthroline, 1-phenyl­pyrazole, inter­molecular hydrogen bonding

## Abstract

The cationic complex in the title compound, [Ir(C_9_H_7_N_2_)_2_(C_12_H_8_N_2_)]PF_6_, comprises two phenyl­pyrazole (ppz) cyclo­metallating ligands and one 1,10-phenanthroline (phen) ancillary ligand. The asymmetric unit consists of one [Ir(ppz)_2_(phen)]^+^ cation and one [PF_6_]^−^ counter-ion. The central Ir^III^ ion is six-coordinated by two N atoms and two C atoms from the two ppz ligands as well as by two N atoms from the phen ligand within a distorted octa­hedral C_2_N_4_ coordination set. In the crystal structure, the [Ir(ppz)_2_(phen)]^+^ cations and PF_6_^−^ counter-ions are connected with each other through weak inter­molecular C—H⋯F hydrogen bonds. Additional C—H⋯*π* inter­actions between the rings of neighbouring cations consolidate the three-dimensional network. Electron density associated with additional disordered solvent mol­ecules inside cavities of the structure was removed with the SQUEEZE procedure in *PLATON* [Spek (2015 ▸). *Acta Cryst*. C**71**, 9–18]. The given chemical formula and other crystal data do not take into account the unknown solvent mol­ecule(s). The title compound has a different space-group symmetry (*C*2/*c*) from its solvatomorph (*P*2_1_/*c*) comprising 1.5CH_2_Cl_2_ solvent mol­ecules per ion pair.

## Abstract 2

The cationic cyclo­metallated iridium(III) complex [Ir(C_9_H_7_N_2_)_2_(C_12_H_8_N_2_)](PF_6_) has been synthesized and crystallized by the inter-diffusion method. It contains an unknown number of solvent mol­ecules and has a different space-group symmetry (*C*2/*c*) structure than its solvatomorph (*P*2_1_/*c*).

## Chemical context   

Cyclo­metallated iridium(III) complexes have found applications in electroluminescent instruments such as sensors and light-emitting devices and in photocatalysis because of their high emission efficiencies, photo/thermal stabilities and easy tunability of the emission wavelength (Zhao *et al.*, 2010 ▸; Shan *et al.*, 2012 ▸). In this regard, a variety of cyclo­metallated iridium complexes have been reported and most of them have potential for the aforementioned applications (Flamigni *et al.*, 2008 ▸; Li *et al.*, 2011 ▸). The properties of iridium complexes can be tuned by rational design of either the cyclo­metallating or ancillary ligands (Chen *et al.*, 2010 ▸; Goswami *et al.*, 2014 ▸; Radwan *et al.*, 2015 ▸; Congrave *et al.*, 2017 ▸). Among numerous organic conjugate ligands, the cyclo­metallating ligand 1-phenyl­pyrazole (ppz) is known for its high triplet energy (Schlegel & Skancke, 1993 ▸). Consequently, some bis-cyclo­metallated Ir^III^ complexes with ppz ligands have been synthesized that exhibit high energy phospho­rescence (Sajoto *et al.*, 2005 ▸).

On the other hand, ancillary ligands with strong conjugated system such as 1,10-phenanthroline (phen) can also enhance the degree of delocalized *π*-electrons of cyclo­metallated iridium(III) complex systems through the inter­action between the *d* orbitals of the transition metal and the *π*-electron orbitals of the organic conjugated system (Liu *et al.*, 2018 ▸). This way, the high degree of delocalized *π*-electrons can increase the luminescent properties of Ir^III^ complexes (Choy *et al.*, 2014 ▸). In this context, we report herein the synthesis and crystal structure of the cyclo­metallated iridium(III) complex, [Ir(ppz)_2_(phen)][PF_6_], which contains an unknown number of solvent mol­ecules.
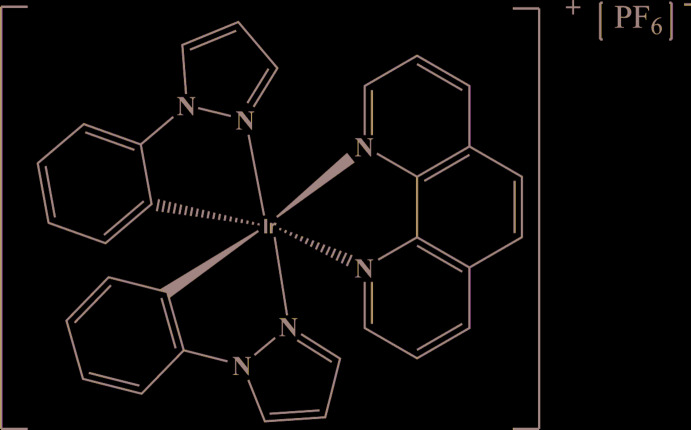


## Structural commentary   

**Figure 1:**
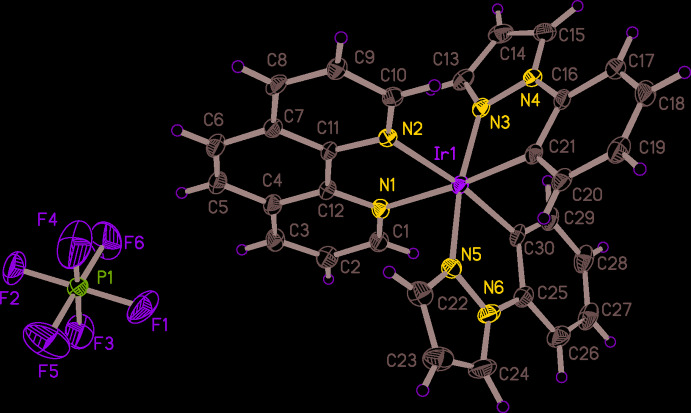
The structures of the mol­ecular entities in the title compound, with displacement ellipsoids drawn at the 30% probability level. H atoms are represented by spheres of arbitrary radius.

The asymmetric unit of the title complex consists of one [Ir(ppz)_2_(phen)]^+^ cation and one PF_6_^−^ counter-ion (Fig. 1 ▸). The iridium(III) atom is six-coordinated by four nitro­gen atoms and two carbon atoms within an octa­hedral [N_4_C_2_] coordination set. The axial positions are occupied by two nitro­gen atoms (N3, N5) from two ppz ligands, while the equatorial plane is composed of two N atoms from the phen ligand (N1, N2) and two C atoms from two ppz ligands (C21, C30).

The bond lengths and angles related to the coordinating carbon and nitro­gen atoms are normal and correspond to literature values. The average Ir—C bond length is 2.018 (5) Å, a typical value for the distance between an Ir^III^ and a C atom originating from a ppz ligand (Adamovich *et al.*, 2019 ▸). There are two different Ir—N bond types in the cation of the title compound: the average Ir—N_C^N_ (C^N refers to the ppz ligand) bond length is 2.023 (2) Å, whereas the value for the Ir—N_N^N_ (N^N refers to the phen ligand) bond is much longer at 2.141 (8) Å. The bond angles around the Ir^III^ atom involving *cis*-arranged ligand atoms deviate clearly from 90° and range from 78.06 (15)° (the bite angle of the phen ligand) to 99.24 (17)°, except for C21—Ir1—C30 with a value of 89.44 (19)°, which correspond to a relatively low distortion from an ideal octa­hedral coordination polyhedron. The bond angles along the axes of the pseudo-octa­hedral coordination figure are 171.64 (16), 173.09 (18) and 173.97 (17)° for N3—Ir—N5, C30—Ir—N2 and C21—Ir—N1, respectively.

## Supra­molecular features   

**Figure 2:**
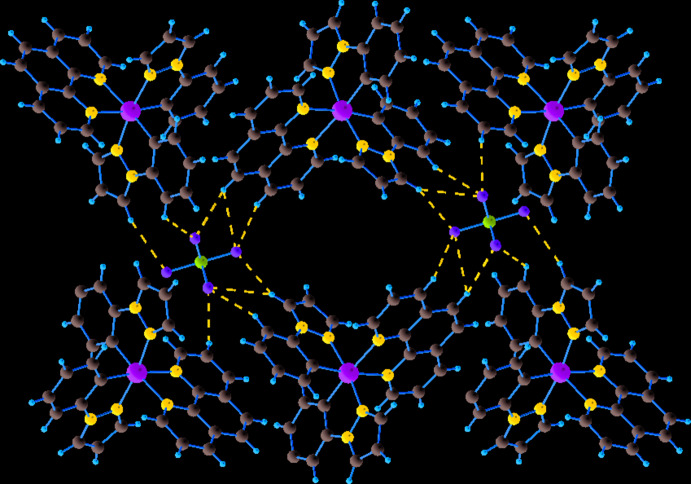
C—H⋯F hydrogen bonding inter­actions between complex cations and counter-ions (shown as dashed lines).

**Figure 3:**
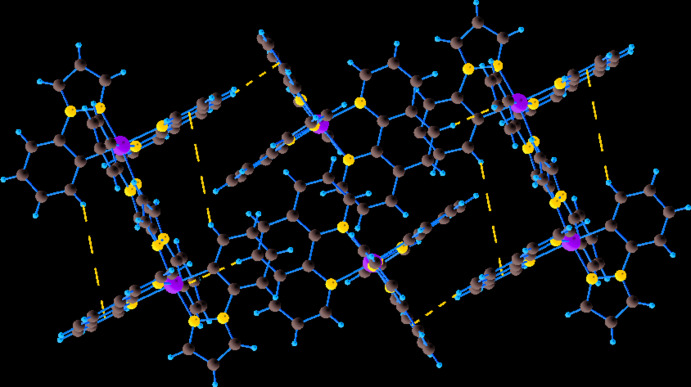
C—H⋯*π* inter­actions in the title structure (shown as dashed lines).

**Figure 4:**
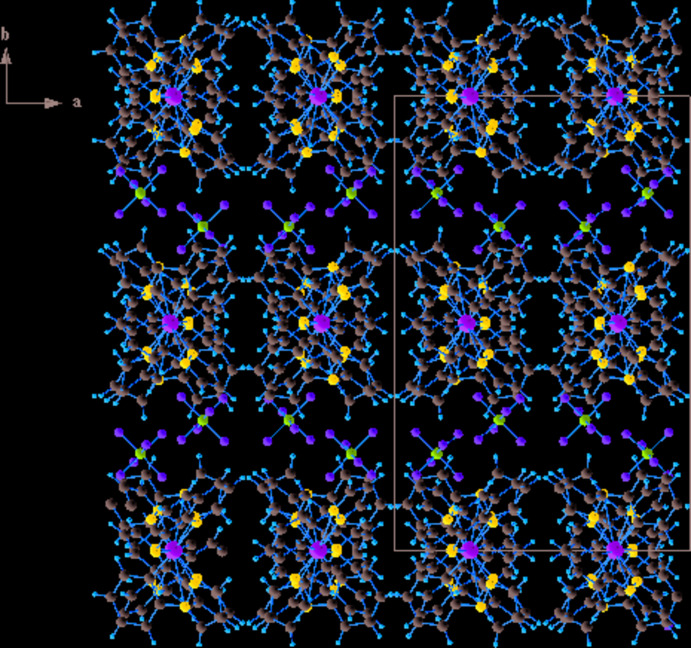
A packing diagram of the title compound viewed along the *c* axis, showing the porous structure with different cavities for the unknown solvent mol­ecules.

In the crystal structure, the complex cations are linked to the PF_6_^−^ counter-ions by six C—H⋯F inter­actions (Table 1 ▸, Fig. 2 ▸), leading to the formation of a three-dimensional supra­molecular network. In addition, there are also C—H⋯*π* inter­actions between the [Ir(ppz)_2_(phen)]^+^ cations, involving the centroids of one of the pyrazole rings and of a phenyl ring (Table 1 ▸, Fig. 3 ▸). As can be seen in Fig. 4 ▸, the packing of the components leads to voids that are large enough to host solvent mol­ecules of an unknown nature.

## Database survey   

A search of the Cambridge Structural Database (CSD, Version 5.39, update November 2017; Groom *et al.*, 2016 ▸) for complexes containing the iridium(III) ion with ppz ligand fragments yielded 36 hits. Among the 36 structures, only four contain auxiliary phen ligands or derivatives thereof. From these, one compound (JUPTIZ; Howarth *et al.*, 2015 ▸) matches the title compound, but crystallizes in the space group *P*2_1_/*c* and contains two mol­ecular ion pairs in the asymmetric unit in contrast to the title compound, which crystallizes in space group *C*2/*c* with one ion pair in the asymmetric unit. Bond lengths and angles in the corresponding [Ir(ppz)_2_(phen)]^+^ cations are very similar. In both structure refinements, the contributions of solvent mol­ecules were not considered; for JUPTIZ, 1.5 CH_2_Cl_2_ solvent mol­ecules were estimated per ion pair, but in the title structure the number and nature of solvent mol­ecule(s) remains unknown. Hence JUPTIZ is a solvatomorph of the title compound. The three other structures comprise derivatives of the phen ligand, *viz*. JUPTEV/JUPTAR (Howarth *et al.*, 2015 ▸) and DUCWOZ (Shan *et al.*, 2012 ▸).

## Synthesis and crystallization   

The organometallated iridium(III) dimer, [Ir(*μ*-Cl)(ppz)_2_]_2_ (ppz = 1-phenyl­pyrazole), was prepared according to a literature protocol (Kwon *et al.*, 2005 ▸) by heating IrCl_3_·3H_2_O (1 equiv.) and 1-phenyl­pyrazole (2.3 equiv.) in a mixed solution of 2-eth­oxy­ethanol and water (*v*/*v* = 3:1) at 408 K.

The title compound was synthesized from the reaction of [Ir(*μ*-Cl)(ppz)_2_]_2_ and 1,10-phenanthroline in a mixed solution of di­chloro­methane (CH_2_Cl_2_) and methanol (MeOH) (*v*/*v* = 2:1) at 358 K with KPF_6_ as a source for the PF_6_^−^ counter-ion. The mixture was dried under vacuum and separated by column chromatography on silica gel with CH_2_Cl_2_/petroleum ether (*v*/*v* = 4:1) as eluent. A pure product of the cyclo­metalated iridium(III) complex was obtained as a dark-yellow solid. Elemental analysis for C_30_H_22_F_6_IrN_6_P (calculated; found): C (44.83; 45.26); H (2.76, 2.73); N (10.46, 10.39)%.

Single crystals of the title compound were grown by inter-diffusion reaction between *n*-hexane and a di­chloro­methane solution of the pure solid with CH_2_Cl_2_/hexane (*v*/*v* = 1/1) as buffer solution at room temperature for 7 d (Nie *et al.*, 2019 ▸). In should be noted that the di­chloro­methane sesquisolvate of [Ir(ppz)_2_(phen)](PF_6_) (JUPTIZ) was obtained by reacting [Ir(*μ*-Cl)(ppz)_2_]_2_ with 1,10-phenanthroline under microwave irradiation for 30 min. at 373 K (Howarth *et al.*, 2015 ▸).

## Refinement   

Crystal data, data collection and structure refinement details are summarized in Table 2 ▸. Carbon-bound H atoms were placed in calculated positions (C—H = 0.93 Å) and were included in the refinement in the riding-model approximation, with *U*_iso_(H) set to 1.2*U*_eq_(C). The contribution of the missing solvent mol­ecules to the diffraction pattern was subtracted from the reflection data by the SQUEEZE method (Spek, 2015 ▸) as implemented in *PLATON* (Spek, 2020 ▸). The solvent-accessible volume in the structure of the title compound as calculated by *PLATON* is 1136.1 Å^3^ (17.7%).

## Supplementary Material

Crystal structure: contains datablock(s) I. DOI: 10.1107/S2056989020005861/wm5544sup1.cif

Structure factors: contains datablock(s) I. DOI: 10.1107/S2056989020005861/wm5544Isup2.hkl

CCDC reference: 1856996

Additional supporting information:  crystallographic information; 3D view; checkCIF report

## Figures and Tables

**Table 1:** Hydrogen-bond geometry (Å, °) *Cg*4 and *Cg*9 are the centroids of rings N3/N4/C15–C13 and C16–C21, respectively.

*D*—H⋯*A*	*D*—H	H⋯*A*	*D*⋯*A*	*D*—H⋯*A*
C2—H2*A*⋯F6^i^	0.93	2.57	3.184 (7)	124
C9—H9*A*⋯F3^ii^	0.93	2.49	3.024 (7)	117
C17—H17*A*⋯F4^iii^	0.93	2.36	2.977 (8)	124
C23—H23*A*⋯F2^iv^	0.93	2.53	3.383 (8)	152
C24—H24*A*⋯F1^v^	0.93	2.48	3.368 (8)	161
C26—H26*A*⋯F5^v^	0.93	2.47	3.348 (9)	158
C6—H6*A*⋯*Cg*9^vi^	0.93	2.58	3.501 (6)	173
C29—H29*A*⋯*Cg*4	0.93	2.98	3.688 (7)	134

Symmetry codes: (i) 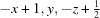; (ii) 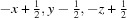; (iii) 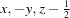; (iv) 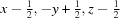; (v) 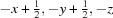; (vi) 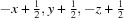.

**Table 2:** Experimental details

Crystal data	
Chemical formula	[Ir(C_9_H_7_N_2_)_2_(C_12_H_8_N_2_)]PF_6_
*M*_r_	803.70
Crystal system, space group	Monoclinic, *C*2/*c*
Temperature (K)	293
*a*, *b*, *c* (Å)	14.976 (3), 22.818 (5), 18.850 (4)
β (°)	95.98 (3)
*V* (Å^3^)	6406 (2)
*Z*	8
Radiation type	Mo *K*α
μ (mm^−1^)	4.28
Crystal size (mm)	0.25 × 0.22 × 0.20

Data collection	
Diffractometer	Bruker APEXII CCD
Absorption correction	Multi-scan (*SADABS*; Krause *et al.*, 2015 ▸)
No. of measured, independent and observed [*I* > 2σ(*I*)] reflections	29484, 6280, 5077
*R*_int_	0.039
(sin θ/λ)_max_ (Å^−1^)	0.618

Refinement	
*R*[*F* ^2^ > 2σ(*F* ^2^)], *wR*(*F* ^2^), *S*	0.039, 0.094, 1.11
No. of reflections	6280
No. of parameters	397
H-atom treatment	H-atom parameters constrained
Δρ_max_, Δρ_min_ (e Å^−3^)	1.25, −0.96

Computer programs: *APEX3* and *SAINT* (Bruker, 2016 ▸), *SHELXT* (Sheldrick, 2015*a* ▸), *SHELXL* (Sheldrick, 2015*b* ▸), *PLATON* (Spek, 2020 ▸) and *DIAMOND* (Brandenburg & Putz, 2016 ▸), *publCIF* (Westrip, 2010 ▸).

## supplementary crystallographic information

### Crystal data

**Table:**

[Ir(C_9_H_7_N_2_)_2_(C_12_H_8_N_2_)]PF_6_	*F*(000) = 3120
*M**_r_* = 803.70	*D*_x_ = 1.667 Mg m^−^^3^
Monoclinic, *C*2/*c*	Mo *K*α radiation, λ = 0.71073 Å
*a* = 14.976 (3) Å	Cell parameters from 10866 reflections
*b* = 22.818 (5) Å	θ = 3.0–26.0°
*c* = 18.850 (4) Å	µ = 4.28 mm^−^^1^
β = 95.98 (3)°	*T* = 293 K
*V* = 6406 (2) Å^3^	Block, red
*Z* = 8	0.25 × 0.22 × 0.20 mm

### Data collection

**Table:**

Bruker APEXII CCD diffractometer	5077 reflections with *I* > 2σ(*I*)
phi and ω scans	*R*_int_ = 0.039
Absorption correction: multi-scan (SADABS; Krause *et al.*, 2015)	θ_max_ = 26.0°, θ_min_ = 3.0°
	*h* = −15→18
29484 measured reflections	*k* = −28→25
6280 independent reflections	*l* = −23→23

### Refinement

**Table:**

Refinement on *F*^2^	0 restraints
Least-squares matrix: full	Hydrogen site location: inferred from neighbouring sites
*R*[*F*^2^ > 2σ(*F*^2^)] = 0.039	H-atom parameters constrained
*wR*(*F*^2^) = 0.094	*w* = 1/[σ^2^(*F*_o_^2^) + (0.0321*P*)^2^ + 39.6215*P*] where *P* = (*F*_o_^2^ + 2*F*_c_^2^)/3
*S* = 1.11	(Δ/σ)_max_ < 0.001
6280 reflections	Δρ_max_ = 1.25 e Å^−^^3^
397 parameters	Δρ_min_ = −0.96 e Å^−^^3^

### Special details

**Table:**

Geometry. All esds (except the esd in the dihedral angle between two l.s. planes) are estimated using the full covariance matrix. The cell esds are taken into account individually in the estimation of esds in distances, angles and torsion angles; correlations between esds in cell parameters are only used when they are defined by crystal symmetry. An approximate (isotropic) treatment of cell esds is used for estimating esds involving l.s. planes.

### Fractional atomic coordinates and isotropic or equivalent isotropic displacement parameters (Å^2^)

**Table:**

	*x*	*y*	*z*	*U*_iso_*/*U*_eq_	
Ir1	0.25544 (2)	−0.00046 (2)	0.00566 (2)	0.02570 (9)	
N1	0.3343 (3)	0.06189 (18)	0.0705 (2)	0.0289 (10)	
N2	0.1946 (3)	−0.00380 (17)	0.1030 (2)	0.0254 (9)	
N3	0.3270 (3)	−0.07374 (19)	0.0323 (2)	0.0289 (10)	
N4	0.2938 (3)	−0.12375 (18)	−0.0004 (2)	0.0317 (10)	
N5	0.1815 (3)	0.06810 (19)	−0.0356 (2)	0.0323 (10)	
N6	0.2036 (3)	0.08734 (19)	−0.1000 (2)	0.0362 (11)	
C1	0.4018 (4)	0.0938 (2)	0.0528 (3)	0.0362 (13)	
H1A	0.4237	0.0871	0.0091	0.043*	
C2	0.4418 (4)	0.1377 (3)	0.0976 (3)	0.0416 (14)	
H2A	0.4888	0.1598	0.0831	0.050*	
C3	0.4118 (4)	0.1478 (2)	0.1623 (3)	0.0397 (14)	
H3A	0.4386	0.1764	0.1926	0.048*	
C4	0.3391 (4)	0.1142 (2)	0.1827 (3)	0.0348 (12)	
C5	0.3015 (4)	0.1213 (3)	0.2491 (3)	0.0408 (14)	
H5A	0.3250	0.1498	0.2811	0.049*	
C6	0.2328 (4)	0.0877 (2)	0.2662 (3)	0.0378 (13)	
H6A	0.2101	0.0930	0.3099	0.045*	
C7	0.1946 (3)	0.0440 (2)	0.2182 (3)	0.0296 (11)	
C8	0.1236 (4)	0.0068 (2)	0.2333 (3)	0.0334 (12)	
H8A	0.0989	0.0101	0.2764	0.040*	
C9	0.0914 (4)	−0.0341 (2)	0.1843 (3)	0.0326 (12)	
H9A	0.0450	−0.0589	0.1940	0.039*	
C10	0.1287 (3)	−0.0384 (2)	0.1192 (3)	0.0300 (11)	
H10A	0.1063	−0.0665	0.0863	0.036*	
C11	0.2288 (3)	0.0364 (2)	0.1529 (2)	0.0264 (11)	
C12	0.3025 (3)	0.0718 (2)	0.1349 (3)	0.0277 (11)	
C13	0.4046 (4)	−0.0891 (3)	0.0694 (3)	0.0367 (13)	
H13A	0.4422	−0.0638	0.0973	0.044*	
C14	0.4207 (4)	−0.1483 (3)	0.0602 (3)	0.0445 (15)	
H14A	0.4698	−0.1697	0.0805	0.053*	
C15	0.3503 (4)	−0.1691 (3)	0.0154 (3)	0.0417 (14)	
H15A	0.3426	−0.2073	−0.0011	0.050*	
C16	0.2090 (4)	−0.1185 (2)	−0.0414 (3)	0.0302 (12)	
C17	0.1642 (4)	−0.1663 (3)	−0.0724 (3)	0.0422 (14)	
H17A	0.1892	−0.2036	−0.0679	0.051*	
C18	0.0812 (4)	−0.1576 (3)	−0.1106 (3)	0.0465 (16)	
H18A	0.0498	−0.1892	−0.1320	0.056*	
C19	0.0454 (4)	−0.1023 (3)	−0.1166 (3)	0.0426 (14)	
H19A	−0.0103	−0.0966	−0.1424	0.051*	
C20	0.0912 (4)	−0.0547 (2)	−0.0845 (3)	0.0332 (12)	
H20A	0.0653	−0.0176	−0.0887	0.040*	
C21	0.1757 (3)	−0.0614 (2)	−0.0458 (3)	0.0287 (11)	
C22	0.1125 (4)	0.0995 (3)	−0.0200 (3)	0.0428 (14)	
H22A	0.0840	0.0954	0.0213	0.051*	
C23	0.0886 (4)	0.1395 (3)	−0.0738 (4)	0.0528 (17)	
H23A	0.0421	0.1666	−0.0757	0.063*	
C24	0.1476 (5)	0.1307 (3)	−0.1235 (3)	0.0515 (17)	
H24A	0.1487	0.1511	−0.1661	0.062*	
C25	0.2783 (4)	0.0599 (2)	−0.1271 (3)	0.0358 (13)	
C26	0.3100 (4)	0.0767 (3)	−0.1898 (3)	0.0478 (16)	
H26A	0.2833	0.1077	−0.2163	0.057*	
C27	0.3814 (5)	0.0474 (3)	−0.2130 (3)	0.0514 (17)	
H27A	0.4039	0.0586	−0.2551	0.062*	
C28	0.4198 (5)	0.0012 (3)	−0.1737 (3)	0.0514 (17)	
H28A	0.4680	−0.0188	−0.1896	0.062*	
C29	0.3863 (4)	−0.0159 (3)	−0.1097 (3)	0.0371 (13)	
H29A	0.4128	−0.0472	−0.0839	0.045*	
C30	0.3144 (4)	0.0132 (2)	−0.0845 (3)	0.0308 (12)	
P1	0.35528 (11)	0.28676 (7)	0.33205 (8)	0.0404 (4)	
F1	0.3359 (4)	0.2677 (2)	0.2511 (2)	0.105 (2)	
F2	0.3773 (4)	0.3061 (2)	0.4122 (2)	0.0877 (15)	
F3	0.4239 (4)	0.3339 (2)	0.3101 (3)	0.0978 (17)	
F4	0.2835 (5)	0.2436 (3)	0.3520 (4)	0.152 (3)	
F5	0.2829 (4)	0.3381 (3)	0.3221 (4)	0.140 (2)	
F6	0.4276 (5)	0.2399 (3)	0.3409 (3)	0.147 (3)	

### Atomic displacement parameters (Å^2^)

**Table:**

	*U*^11^	*U*^22^	*U*^33^	*U*^12^	*U*^13^	*U*^23^
Ir1	0.02768 (12)	0.02890 (14)	0.02103 (12)	−0.00258 (8)	0.00499 (8)	0.00069 (8)
N1	0.029 (2)	0.029 (2)	0.028 (2)	−0.0065 (19)	0.0014 (19)	−0.0035 (18)
N2	0.026 (2)	0.026 (2)	0.024 (2)	−0.0003 (17)	0.0036 (17)	0.0014 (17)
N3	0.033 (2)	0.034 (2)	0.022 (2)	−0.0015 (19)	0.0107 (19)	−0.0002 (18)
N4	0.042 (3)	0.029 (2)	0.025 (2)	−0.001 (2)	0.008 (2)	0.0023 (18)
N5	0.036 (3)	0.029 (2)	0.031 (2)	−0.005 (2)	0.002 (2)	−0.0001 (19)
N6	0.042 (3)	0.037 (3)	0.029 (2)	−0.006 (2)	0.000 (2)	0.010 (2)
C1	0.040 (3)	0.037 (3)	0.032 (3)	−0.010 (3)	0.004 (2)	−0.001 (2)
C2	0.037 (3)	0.046 (4)	0.042 (4)	−0.012 (3)	0.005 (3)	0.000 (3)
C3	0.040 (3)	0.042 (3)	0.035 (3)	−0.010 (3)	−0.005 (3)	−0.008 (3)
C4	0.039 (3)	0.037 (3)	0.028 (3)	−0.005 (2)	0.000 (2)	−0.003 (2)
C5	0.053 (4)	0.043 (3)	0.026 (3)	−0.005 (3)	0.002 (3)	−0.004 (2)
C6	0.043 (3)	0.046 (3)	0.026 (3)	−0.004 (3)	0.008 (2)	−0.003 (2)
C7	0.033 (3)	0.031 (3)	0.024 (3)	0.004 (2)	0.003 (2)	0.001 (2)
C8	0.037 (3)	0.041 (3)	0.023 (3)	0.002 (2)	0.013 (2)	0.007 (2)
C9	0.035 (3)	0.037 (3)	0.027 (3)	0.000 (2)	0.009 (2)	0.001 (2)
C10	0.031 (3)	0.030 (3)	0.029 (3)	−0.003 (2)	0.007 (2)	0.003 (2)
C11	0.031 (3)	0.029 (3)	0.019 (2)	0.000 (2)	0.000 (2)	−0.001 (2)
C12	0.027 (3)	0.034 (3)	0.022 (3)	0.001 (2)	0.001 (2)	−0.001 (2)
C13	0.029 (3)	0.052 (4)	0.029 (3)	0.000 (3)	0.001 (2)	0.007 (3)
C14	0.047 (4)	0.045 (4)	0.042 (4)	0.014 (3)	0.008 (3)	0.014 (3)
C15	0.055 (4)	0.034 (3)	0.038 (3)	0.007 (3)	0.014 (3)	0.008 (3)
C16	0.039 (3)	0.033 (3)	0.019 (3)	−0.005 (2)	0.006 (2)	−0.002 (2)
C17	0.065 (4)	0.035 (3)	0.027 (3)	−0.008 (3)	0.009 (3)	0.000 (2)
C18	0.059 (4)	0.046 (4)	0.033 (3)	−0.028 (3)	0.000 (3)	−0.005 (3)
C19	0.041 (3)	0.057 (4)	0.029 (3)	−0.014 (3)	0.000 (3)	0.002 (3)
C20	0.034 (3)	0.043 (3)	0.022 (3)	−0.008 (2)	0.005 (2)	0.001 (2)
C21	0.034 (3)	0.035 (3)	0.019 (2)	−0.009 (2)	0.010 (2)	0.000 (2)
C22	0.033 (3)	0.044 (3)	0.051 (4)	0.010 (3)	0.004 (3)	−0.001 (3)
C23	0.042 (4)	0.051 (4)	0.063 (5)	0.007 (3)	−0.009 (3)	0.011 (3)
C24	0.062 (4)	0.045 (4)	0.044 (4)	0.001 (3)	−0.011 (3)	0.018 (3)
C25	0.038 (3)	0.041 (3)	0.028 (3)	−0.007 (3)	0.001 (2)	0.002 (2)
C26	0.061 (4)	0.047 (4)	0.035 (3)	−0.019 (3)	0.004 (3)	0.011 (3)
C27	0.058 (4)	0.071 (5)	0.028 (3)	−0.026 (4)	0.016 (3)	−0.002 (3)
C28	0.051 (4)	0.072 (5)	0.033 (3)	−0.017 (3)	0.015 (3)	−0.009 (3)
C29	0.038 (3)	0.050 (3)	0.025 (3)	−0.008 (3)	0.012 (2)	−0.005 (2)
C30	0.032 (3)	0.044 (3)	0.017 (2)	−0.012 (2)	0.004 (2)	−0.004 (2)
P1	0.0506 (9)	0.0377 (8)	0.0325 (8)	−0.0028 (7)	0.0030 (7)	0.0021 (6)
F1	0.163 (5)	0.105 (4)	0.041 (3)	−0.067 (4)	−0.014 (3)	0.005 (2)
F2	0.127 (4)	0.095 (3)	0.041 (2)	−0.017 (3)	0.009 (2)	−0.019 (2)
F3	0.128 (4)	0.083 (3)	0.086 (3)	−0.054 (3)	0.027 (3)	−0.018 (3)
F4	0.198 (7)	0.158 (6)	0.116 (5)	−0.125 (5)	0.084 (5)	−0.035 (4)
F5	0.103 (4)	0.108 (4)	0.200 (7)	0.049 (4)	−0.023 (4)	0.011 (4)
F6	0.183 (7)	0.146 (5)	0.104 (5)	0.135 (5)	−0.024 (4)	−0.014 (4)

### Geometric parameters (Å, º)

**Table:**

Ir1—C21	2.016 (5)	C11—C12	1.437 (7)
Ir1—C30	2.019 (5)	C13—C14	1.388 (8)
Ir1—N3	2.021 (4)	C13—H13A	0.9300
Ir1—N5	2.025 (4)	C14—C15	1.365 (9)
Ir1—N2	2.133 (4)	C14—H14A	0.9300
Ir1—N1	2.148 (4)	C15—H15A	0.9300
N1—C1	1.317 (7)	C16—C17	1.378 (7)
N1—C12	1.367 (6)	C16—C21	1.394 (7)
N2—C10	1.324 (6)	C17—C18	1.383 (9)
N2—C11	1.374 (6)	C17—H17A	0.9300
N3—C13	1.339 (7)	C18—C19	1.370 (9)
N3—N4	1.366 (6)	C18—H18A	0.9300
N4—C15	1.349 (7)	C19—C20	1.390 (8)
N4—C16	1.420 (7)	C19—H19A	0.9300
N5—C22	1.315 (7)	C20—C21	1.401 (7)
N5—N6	1.364 (6)	C20—H20A	0.9300
N6—C24	1.341 (7)	C22—C23	1.383 (8)
N6—C25	1.422 (7)	C22—H22A	0.9300
C1—C2	1.402 (8)	C23—C24	1.369 (9)
C1—H1A	0.9300	C23—H23A	0.9300
C2—C3	1.364 (8)	C24—H24A	0.9300
C2—H2A	0.9300	C25—C26	1.374 (8)
C3—C4	1.418 (8)	C25—C30	1.408 (8)
C3—H3A	0.9300	C26—C27	1.372 (9)
C4—C12	1.395 (7)	C26—H26A	0.9300
C4—C5	1.434 (8)	C27—C28	1.378 (9)
C5—C6	1.350 (8)	C27—H27A	0.9300
C5—H5A	0.9300	C28—C29	1.409 (8)
C6—C7	1.425 (7)	C28—H28A	0.9300
C6—H6A	0.9300	C29—C30	1.391 (8)
C7—C11	1.393 (7)	C29—H29A	0.9300
C7—C8	1.413 (7)	P1—F6	1.520 (5)
C8—C9	1.364 (7)	P1—F4	1.534 (5)
C8—H8A	0.9300	P1—F3	1.572 (5)
C9—C10	1.404 (7)	P1—F2	1.575 (4)
C9—H9A	0.9300	P1—F1	1.584 (5)
C10—H10A	0.9300	P1—F5	1.593 (5)
			
C21—Ir1—C30	89.44 (19)	N3—C13—C14	110.0 (5)
C21—Ir1—N3	79.7 (2)	N3—C13—H13A	125.0
C30—Ir1—N3	94.0 (2)	C14—C13—H13A	125.0
C21—Ir1—N5	94.3 (2)	C15—C14—C13	106.3 (5)
C30—Ir1—N5	80.0 (2)	C15—C14—H14A	126.8
N3—Ir1—N5	171.64 (16)	C13—C14—H14A	126.8
C21—Ir1—N2	96.00 (17)	N4—C15—C14	107.3 (5)
C30—Ir1—N2	173.09 (18)	N4—C15—H15A	126.3
N3—Ir1—N2	91.16 (15)	C14—C15—H15A	126.3
N5—Ir1—N2	95.29 (16)	C17—C16—C21	123.9 (5)
C21—Ir1—N1	173.97 (17)	C17—C16—N4	122.2 (5)
C30—Ir1—N1	96.56 (18)	C21—C16—N4	114.0 (4)
N3—Ir1—N1	99.24 (17)	C16—C17—C18	118.6 (6)
N5—Ir1—N1	87.33 (17)	C16—C17—H17A	120.7
N2—Ir1—N1	78.06 (15)	C18—C17—H17A	120.7
C1—N1—C12	118.8 (4)	C19—C18—C17	119.8 (5)
C1—N1—Ir1	127.8 (4)	C19—C18—H18A	120.1
C12—N1—Ir1	113.1 (3)	C17—C18—H18A	120.1
C10—N2—C11	118.2 (4)	C18—C19—C20	120.9 (6)
C10—N2—Ir1	128.0 (3)	C18—C19—H19A	119.6
C11—N2—Ir1	113.8 (3)	C20—C19—H19A	119.6
C13—N3—N4	105.8 (4)	C19—C20—C21	121.2 (5)
C13—N3—Ir1	139.0 (4)	C19—C20—H20A	119.4
N4—N3—Ir1	114.8 (3)	C21—C20—H20A	119.4
C15—N4—N3	110.5 (5)	C16—C21—C20	115.7 (5)
C15—N4—C16	133.6 (5)	C16—C21—Ir1	115.2 (4)
N3—N4—C16	116.0 (4)	C20—C21—Ir1	129.2 (4)
C22—N5—N6	107.0 (5)	N5—C22—C23	110.2 (6)
C22—N5—Ir1	138.2 (4)	N5—C22—H22A	124.9
N6—N5—Ir1	114.6 (3)	C23—C22—H22A	124.9
C24—N6—N5	109.3 (5)	C24—C23—C22	105.6 (6)
C24—N6—C25	133.9 (5)	C24—C23—H23A	127.2
N5—N6—C25	116.8 (4)	C22—C23—H23A	127.2
N1—C1—C2	122.2 (5)	N6—C24—C23	107.9 (5)
N1—C1—H1A	118.9	N6—C24—H24A	126.0
C2—C1—H1A	118.9	C23—C24—H24A	126.0
C3—C2—C1	120.0 (5)	C26—C25—C30	123.7 (6)
C3—C2—H2A	120.0	C26—C25—N6	122.8 (5)
C1—C2—H2A	120.0	C30—C25—N6	113.4 (5)
C2—C3—C4	119.0 (5)	C27—C26—C25	119.2 (6)
C2—C3—H3A	120.5	C27—C26—H26A	120.4
C4—C3—H3A	120.5	C25—C26—H26A	120.4
C12—C4—C3	117.5 (5)	C26—C27—C28	119.9 (6)
C12—C4—C5	118.6 (5)	C26—C27—H27A	120.1
C3—C4—C5	123.9 (5)	C28—C27—H27A	120.1
C6—C5—C4	121.5 (5)	C27—C28—C29	120.4 (6)
C6—C5—H5A	119.2	C27—C28—H28A	119.8
C4—C5—H5A	119.2	C29—C28—H28A	119.8
C5—C6—C7	120.7 (5)	C30—C29—C28	121.1 (6)
C5—C6—H6A	119.6	C30—C29—H29A	119.4
C7—C6—H6A	119.6	C28—C29—H29A	119.4
C11—C7—C8	117.1 (5)	C29—C30—C25	115.6 (5)
C11—C7—C6	119.3 (5)	C29—C30—Ir1	129.5 (4)
C8—C7—C6	123.6 (5)	C25—C30—Ir1	114.9 (4)
C9—C8—C7	119.7 (5)	F6—P1—F4	91.8 (5)
C9—C8—H8A	120.2	F6—P1—F3	91.8 (4)
C7—C8—H8A	120.2	F4—P1—F3	176.4 (4)
C8—C9—C10	119.6 (5)	F6—P1—F2	91.0 (3)
C8—C9—H9A	120.2	F4—P1—F2	91.5 (3)
C10—C9—H9A	120.2	F3—P1—F2	89.1 (3)
N2—C10—C9	122.3 (5)	F6—P1—F1	88.2 (3)
N2—C10—H10A	118.9	F4—P1—F1	89.8 (3)
C9—C10—H10A	118.9	F3—P1—F1	89.6 (3)
N2—C11—C7	122.9 (5)	F2—P1—F1	178.4 (3)
N2—C11—C12	117.0 (4)	F6—P1—F5	177.3 (4)
C7—C11—C12	120.0 (4)	F4—P1—F5	90.9 (4)
N1—C12—C4	122.5 (5)	F3—P1—F5	85.5 (3)
N1—C12—C11	117.7 (4)	F2—P1—F5	88.8 (3)
C4—C12—C11	119.8 (4)	F1—P1—F5	92.0 (4)
			
C13—N3—N4—C15	−0.8 (5)	C7—C11—C12—C4	−0.5 (7)
Ir1—N3—N4—C15	174.3 (3)	N4—N3—C13—C14	0.2 (6)
C13—N3—N4—C16	177.8 (4)	Ir1—N3—C13—C14	−173.0 (4)
Ir1—N3—N4—C16	−7.1 (5)	N3—C13—C14—C15	0.4 (6)
C22—N5—N6—C24	0.0 (6)	N3—N4—C15—C14	1.0 (6)
Ir1—N5—N6—C24	177.0 (4)	C16—N4—C15—C14	−177.2 (5)
C22—N5—N6—C25	178.9 (5)	C13—C14—C15—N4	−0.9 (6)
Ir1—N5—N6—C25	−4.1 (6)	C15—N4—C16—C17	3.6 (9)
C12—N1—C1—C2	−0.2 (8)	N3—N4—C16—C17	−174.6 (5)
Ir1—N1—C1—C2	173.0 (4)	C15—N4—C16—C21	−177.5 (5)
N1—C1—C2—C3	0.9 (9)	N3—N4—C16—C21	4.3 (6)
C1—C2—C3—C4	−1.0 (9)	C21—C16—C17—C18	0.2 (8)
C2—C3—C4—C12	0.4 (8)	N4—C16—C17—C18	179.0 (5)
C2—C3—C4—C5	−179.5 (6)	C16—C17—C18—C19	−0.1 (8)
C12—C4—C5—C6	0.8 (9)	C17—C18—C19—C20	−0.3 (9)
C3—C4—C5—C6	−179.3 (6)	C18—C19—C20—C21	0.8 (8)
C4—C5—C6—C7	−0.6 (9)	C17—C16—C21—C20	0.3 (7)
C5—C6—C7—C11	−0.1 (8)	N4—C16—C21—C20	−178.6 (4)
C5—C6—C7—C8	179.0 (5)	C17—C16—C21—Ir1	179.4 (4)
C11—C7—C8—C9	−0.5 (8)	N4—C16—C21—Ir1	0.5 (5)
C6—C7—C8—C9	−179.6 (5)	C19—C20—C21—C16	−0.8 (7)
C7—C8—C9—C10	−0.5 (8)	C19—C20—C21—Ir1	−179.8 (4)
C11—N2—C10—C9	1.9 (7)	N6—N5—C22—C23	0.1 (7)
Ir1—N2—C10—C9	−176.7 (4)	Ir1—N5—C22—C23	−175.8 (4)
C8—C9—C10—N2	−0.2 (8)	N5—C22—C23—C24	−0.1 (7)
C10—N2—C11—C7	−3.0 (7)	N5—N6—C24—C23	−0.1 (7)
Ir1—N2—C11—C7	175.8 (4)	C25—N6—C24—C23	−178.8 (6)
C10—N2—C11—C12	177.8 (4)	C22—C23—C24—N6	0.1 (7)
Ir1—N2—C11—C12	−3.4 (5)	C24—N6—C25—C26	1.4 (10)
C8—C7—C11—N2	2.3 (7)	N5—N6—C25—C26	−177.2 (5)
C6—C7—C11—N2	−178.5 (5)	C24—N6—C25—C30	−176.9 (6)
C8—C7—C11—C12	−178.5 (5)	N5—N6—C25—C30	4.5 (7)
C6—C7—C11—C12	0.7 (8)	C30—C25—C26—C27	−0.7 (9)
C1—N1—C12—C4	−0.5 (8)	N6—C25—C26—C27	−178.9 (5)
Ir1—N1—C12—C4	−174.6 (4)	C25—C26—C27—C28	0.7 (9)
C1—N1—C12—C11	180.0 (5)	C26—C27—C28—C29	−0.3 (9)
Ir1—N1—C12—C11	5.9 (6)	C27—C28—C29—C30	−0.2 (9)
C3—C4—C12—N1	0.3 (8)	C28—C29—C30—C25	0.2 (8)
C5—C4—C12—N1	−179.8 (5)	C28—C29—C30—Ir1	−178.3 (4)
C3—C4—C12—C11	179.9 (5)	C26—C25—C30—C29	0.2 (8)
C5—C4—C12—C11	−0.2 (8)	N6—C25—C30—C29	178.5 (5)
N2—C11—C12—N1	−1.7 (7)	C26—C25—C30—Ir1	179.0 (4)
C7—C11—C12—N1	179.1 (5)	N6—C25—C30—Ir1	−2.7 (6)
N2—C11—C12—C4	178.7 (5)		

### Hydrogen-bond geometry (Å, º)

*Cg*4 and *Cg*9 are the centroids of rings N3/N4/C15–C13 and C16–C21, respectively.

**Table:**

*D*—H···*A*	*D*—H	H···*A*	*D*···*A*	*D*—H···*A*
C2—H2*A*···F6^i^	0.93	2.57	3.184 (7)	124
C9—H9*A*···F3^ii^	0.93	2.49	3.024 (7)	117
C17—H17*A*···F4^iii^	0.93	2.36	2.977 (8)	124
C23—H23*A*···F2^iv^	0.93	2.53	3.383 (8)	152
C24—H24*A*···F1^v^	0.93	2.48	3.368 (8)	161
C26—H26*A*···F5^v^	0.93	2.47	3.348 (9)	158
C6—H6*A*···*Cg*9^vi^	0.93	2.58	3.501 (6)	173
C29—H29*A*···*Cg*4	0.93	2.98	3.688 (7)	134

Symmetry codes: (i) −*x*+1, *y*, −*z*+1/2; (ii) −*x*+1/2, *y*−1/2, −*z*+1/2; (iii) *x*, −*y*, *z*−1/2; (iv) *x*−1/2, −*y*+1/2, *z*−1/2; (v) −*x*+1/2, −*y*+1/2, −*z*; (vi) −*x*+1/2, *y*+1/2, −*z*+1/2.

## References

[bb1] Adamovich, V., Boudreault, P. T., Esteruelas, M. A., Gómez-Bautista, D., López, A. M., Oñate, E. & Tsai, J. Y. (2019). *Organometallics*, **38**, 2738–2747.

[bb2] Brandenburg, K. & Putz, H. (2016). *DIAMOND*. Crystal Impact GbR, Bonn, Germany.

[bb3] Bruker (2016). *APEX3* and *SAINT*. Bruker AXS, Inc., Madison, Wisconsin, USA.

[bb4] Chen, Z. Q., Bian, Z. Q. & Huang, C. H. (2010). *Adv. Mater.* **22**, 1534–1539. 10.1002/adma.200903233 20437503

[bb5] Choy, W. C. H., Chan, W. K. & Yuan, Y. (2014). *Adv. Mater.* **26**, 5368–5399. 10.1002/adma.201306133 25042158

[bb6] Congrave, D. G., Hsu, Y. T., Batsanov, A. S., Beeby, A. & Bryce, M. R. (2017). *Organometallics*, **36**, 981–993.

[bb7] Flamigni, L., Collin, J. P. & Sauvage, J. P. (2008). *Acc. Chem. Res.* **41**, 857–871. 10.1021/ar700282n 18543956

[bb8] Goswami, S., Sengupta, D., Paul, N. D., Mondal, T. K. & Goswami, S. (2014). *Chem. Eur. J.* **20**, 6103–6111. 10.1002/chem.201304369 24682999

[bb9] Groom, C. R., Bruno, I. J., Lightfoot, M. P. & Ward, S. C. (2016). *Acta Cryst.* B**72**, 171–179. 10.1107/S2052520616003954 PMC4822653 27048719

[bb10] Howarth, A. J., Majewski, M. B., Brown, C. M., Lelj, F., Wolf, M. O. & Patrick, B. O. (2015). *Dalton Trans.* **44**, 16272–16279. 10.1039/c5dt02691a 26278384

[bb11] Krause, L., Herbst-Irmer, R., Sheldrick, G. M. & Stalke, D. (2015). *J. Appl. Cryst.* **48**, 3–10. 10.1107/S1600576714022985 PMC4453166 26089746

[bb12] Kwon, T. H., Cho, H. S., Kim, M. K., Kim, J. W., Kim, J. J., Lee, K. H., Park, S. J., Shin, I. S., Kim, H., Shin, D. M., Chung, Y. K. & Hong, J. I. (2005). *Organometallics*, **24**, 1578–1585.

[bb13] Li, F., Zhang, B., Li, X., Jiang, Y., Chen, L., Li, Y. & Sun, L. (2011). *Angew. Chem. Int. Ed.* **50**, 12276–12279. 10.1002/anie.201105044 22028099

[bb14] Liu, B., Monro, S., Lystrom, L., Cameron, C. G., Colón, K., Yin, H., Kilina, S., McFarland, S. A. & Sun, W. (2018). *Inorg. Chem.* **57**, 7122–7131. 10.1021/acs.inorgchem.8b00789 PMC6337720 30091916

[bb15] Nie, Q. Y., Qian, J. & Zhang, C. (2019). *J. Mol. Struct.* **1186**, 434–439.

[bb16] Radwan, Y. K., Maity, A. & Teets, T. S. (2015). *Inorg. Chem.* **54**, 7122–7131. 10.1021/acs.inorgchem.5b01401 26158354

[bb17] Sajoto, T., Djurovich, P. I., Tamayo, A., Yousufuddin, M., Bau, R., Thompson, M. E., Holmes, R. J. & Forrest, S. R. (2005). *Inorg. Chem.* **44**, 7992–8003. 10.1021/ic051296i 16241149

[bb18] Schlegel, H. B. & Skancke, A. (1993). *J. Am. Chem. Soc.* **115**, 7465–7471.

[bb19] Shan, G. G., Li, H. B., Cao, H. T., Zhu, D. X., Su, Z. M. & Liao, Y. (2012). *J. Organomet. Chem.* **713**, 20–26.

[bb20] Sheldrick, G. M. (2015*a*). *Acta Cryst.* C**71**, 3–8.

[bb21] Sheldrick, G. M. (2015*b*). *Acta Cryst.* C**71**, 3–8.

[bb22] Spek, A. L. (2015). *Acta Cryst.* C**71**, 9–18. 10.1107/S2053229614024929 25567569

[bb23] Spek, A. L. (2020). *Acta Cryst.* E**76**, 1–11. 10.1107/S2056989019016244 PMC6944088 31921444

[bb24] Westrip, S. P. (2010). *J. Appl. Cryst.* **43**, 920–925.

[bb25] Zhao, Q., Li, F. Y. & Huang, C. H. (2010). *Chem. Soc. Rev.* **39**, 3007–3030. 10.1039/b915340c 20480067

